# Gas Sensor with Different Morphology of PANI Layer

**DOI:** 10.3390/s23031106

**Published:** 2023-01-18

**Authors:** Jiri Kroutil, Alexandr Laposa, Vojtech Povolny, Ladislav Klimsa, Miroslav Husak

**Affiliations:** 1 Department of Microelectronics, Czech Technical University in Prague, Technicka 2, 166 27 Prague, Czech Republic; 2 Department of Material Analysis, FZU—Institute of Physics of the Czech Academy of Sciences, Na Slovance 1999/2, 182 00 Prague, Czech Republic

**Keywords:** polyaniline, gas sensor, polymer film

## Abstract

This work presents the design of a polymer-film-based sensor for gas detection. Different types of polyaniline are used as active layers. The advantages of resistive sensors with PANI layers are easy preparation and low production cost. At room temperature, polymer films have a high sensitivity to gas concentrations. The developed sensor works on the idea of electrical resistance shifting with gas concentration. Three different polymerization solutions are employed to synthesize the polyaniline (PANI) active layers (aqueous solution, sulfuric acid solution, and acetic acid solution). Active layers are evaluated in a controlled environment for their ability to detect ammonia, carbon monoxide, nitrogen monoxide, acetone, toluene, and relative humidity in synthetic air. PANI layers polymerized in acetic acid solutions exhibit good sensitivity toward ammonia.

## 1. Introduction

The monitoring of gaseous substances, especially toxic substances, is very important in many areas such as automotive, aviation, agriculture, security, health care, defense and security, industry, and environmental monitoring [1,2]. Ammonia, nitrogen oxides, organic volatiles, etc., are among those gasses. Medical applications of gas sensors include respiratory monitoring by analyzing carbon dioxide in exhaled air [3,4]. Active layers with organic materials achieve good sensitivity. Sensor requirements aim to make them smaller and more efficient. Research has focused on increasing resistance to gasses and higher temperatures. Polymer active layers are used in sensor arrays [5,6].

The active layers using conducting polymers are attractive for room temperature operations and easy synthesis. Sensing layers can be synthesized by various methods such as the co-precipitation method, the hydrothermal method, the sol-gel method, and microwave-assisted techniques [7]. Chemical sensors based on conducting polymers such as polyaniline (PANI), polypyrrole (PPy), and polythiophene (PTh) are suitable for their low cost, flexibility, light weight, and simple production [8]. PANI exhibits good stability in a wide range of conductivity and can be easily doped [9]. These features make polyaniline promising for industrial applications and for sensors with high selectivity, fast response times, recovery times, and the option to tailor its particular characteristics [10].

Polyaniline can form various oxidation states (fully oxidized pernigraniline, protoemeraldine, emeraldine, nigraniline, and fully reduced leucoemeraldine). The fully oxidized and reduced polyaniline is not conductive. A conducting emeraldine salt can be obtained, if the oxidation states are slightly doped (especially the emeraldine form) [11].

Low-cost printed sensors for clinical diagnostics and environmental monitoring can take advantage of the polymer active layers, such as low curing temperature (60 °C to 120 °C), thin and smooth films, low-cost production, light weight, and large-area applications [12,13].

Detection of volatile organic compounds (VOCs) using PANI produced via in situ chemical polymerization was reported in [14]. This sensor is operated at room temperature. For the detection of ammonia gas, Safe et al. [15] fabricated a nanotube form of polyaniline with a response of 6% toward 20 ppm of ammonia.

Kumar et al. [16] prepared a PANI-based flexible sensor that detects ammonia in the range of 5–1000 ppm and operates at room temperature. In [17], the optimization of printed polyaniline for gas sensing applications was described. Hydrochloric acid-doped polyaniline was electrochemically synthesized and used as a gas sensor for ammonia in [18]. An ammonia gas sensor based on flexible PANI films was used for the rapid detection of spoilage in protein-rich foods [19]. In addition, polyaniline was doped with metals and metal oxides to increase sensing performance [20,21,22].

In this paper, we presented the design of a gas sensor with polyaniline layers prepared in different polymerization solutions: (i) aqueous solution, (ii) sulfuric acid solution, and (iii) acetic acid solution. Room temperature gas-sensing properties of the fabricated gas sensors to different gas environments (i.e., carbon oxide, carbon dioxide, ammonia, nitrogen dioxide, acetone, toluene, and relative humidity) were studied.

## 2. Materials and Methods

### 2.1. Materials

Isopropyl alcohol 99.8% ((CH_3_)_2_CHOH), acetone 99.5% (CH_3_COCH_3_), sulfuric acid 96% (H_2_SO_4_), acetic acid 99% (CH_3_CO_2_H), and hydrochloric acid 36% (HCl) were purchased from Penta Ltd. (Prague, Czech Republic). Ammonium persulfate 98% ((NH_4_)_2_S_2_O_8_) and aniline hydrochloride 98% (C_6_H_5_NH_2_ HCl) were purchased from Lach-Ner Ltd. (Neratovice, Czech Republic).

### 2.2. Sensor Platform

**Figure 1:**
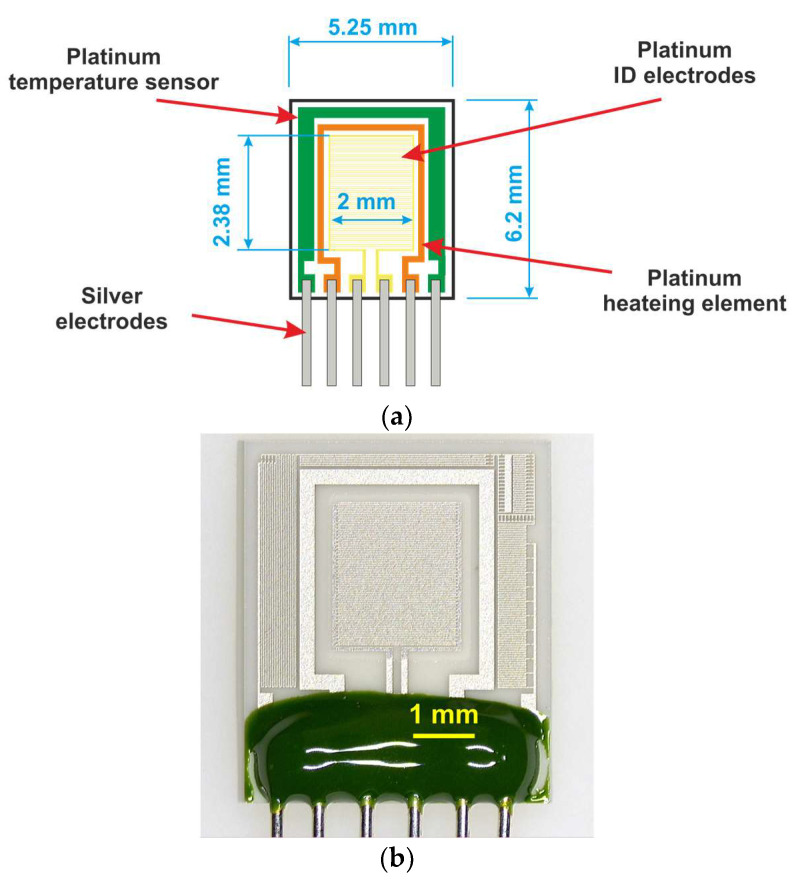
Sensor platform KBI2 Tesla Blatná: (**a**) the schematic diagram with dimensions, (**b**) sensor platform without a sensitive layer.

**Figure 2:**
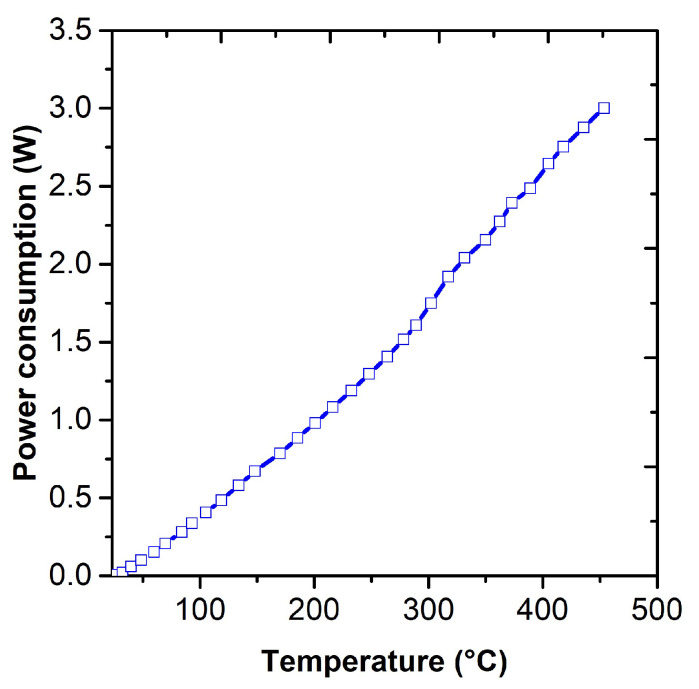
Dependence of the power consumption on the temperature.

The sensor platform KBI2 Tesla Blatná a. s. was used for the study of polyaniline layers (Figure 1). The sensor platform was fabricated by sputtering a thin layer of platinum on a 6.2 mm × 5.25 mm ceramic substrate (Al_2_O_3_), followed by laser trimming to form the structures of the interdigital electrodes, temperature sensor (Pt1000), and heating element. The temperature sensor and the heating element were passivated by an insulating glass layer. Sensitive layers can be deposited on interdigital electrodes by various techniques, such as printing, dipping, screen printing, or immersion. In our study, we used the technique of dipping in a polymerization solution. The temperature element allows a constant temperature to be maintained or performs thermal cycling up to 450 °C. The power consumption as a function of temperature can be seen in Figure 2. At 450 °C, a sensor’s power consumption is 3 W.

Figure 1b shows the structure of the sensor platform. The width and spacing of platinum ID (interdigital) electrodes are 15 µm. There are 80 individual electrode fingers (40 pairs), and the length of each is 2 mm. 

### 2.3. Material Synthesis and Sensor Fabrication

**Figure 3:**
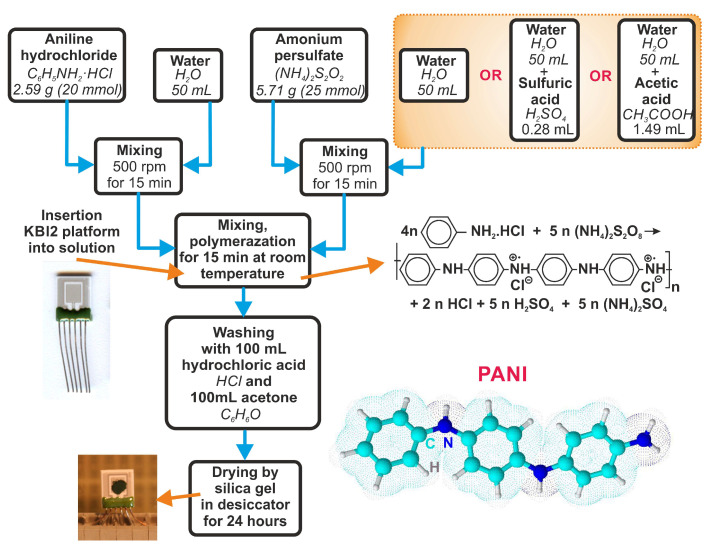
Preparation of various forms of polyaniline.

**Figure 4:**
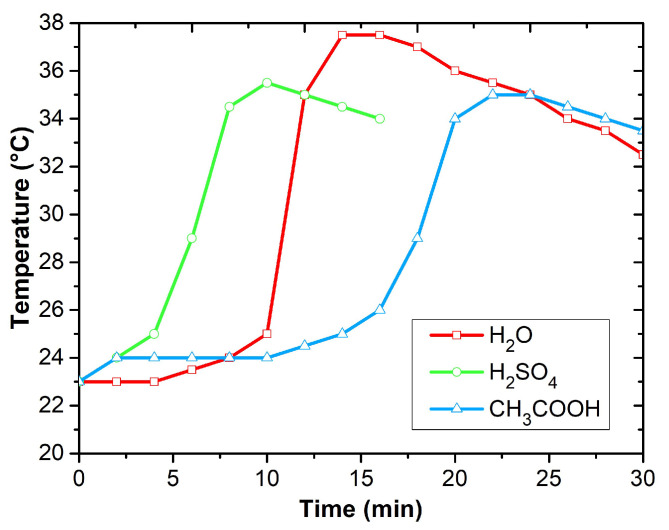
The temperature of the reaction mixture as a function of time.

The sensor platforms were cleaned with acetone and isopropyl alcohol for 15 min prior to the deposition of the sensing layer. At room temperature, 0.2 M aniline hydrochloride was oxidized with 0.25 M ammonium persulfate to produce polyaniline in the form of protonated emeraldine salt (Figure 3). This method was described in [9]. The polymerization was performed in different polymerization solutions: (i) aqueous solution, (ii) sulfuric acid solution, and (iii) acetic acid solution. Since polyaniline synthesis is an exothermic process, the temperature of the reaction mixture was monitored. The effect of temperature on PANI polymerization time is shown in Figure 4. The process of polymerization was finished at 37 °C within 15 min for the aqueous solution, at 36 °C within 10 min for the sulfuric acid solution, and at 35 °C within 25 min for the acetic acid solution. Before drying the sensors on a hotplate at 60 °C for 2 h and then by silica gel in a desiccator for 24 h, they were purified using 0.2 M hydrochloric acid and acetone.

### 2.4. Preparation of the Gas Testing System

**Figure 5:**
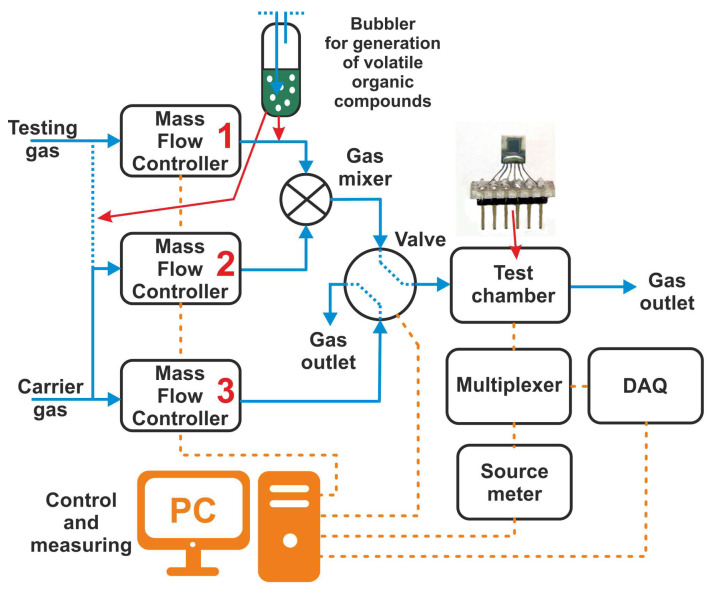
Schematic diagram of the gas sensing characterizations apparatus.

A measuring apparatus was designed for testing the gas sensors (Figure 5). The apparatus allows for the precise adjustment of gas concentrations using Bronkhorst mass flow controllers MFC1 to MFC3 (F-201DV-AAD-22-K in the range 10 mL–500 mL, F-201EV-AAD-22-K in the range 40 mL–2000 mL). Synthetic air (SA: 21% O_2_ and the rest N_2_) is used as a carrier gas, which also serves as a purging gas. The exact concentration of the test gas can be obtained by mixing it with the carrier gas or by diluting the saturated vapor of the desired solvent from the bubbler with dry air. This second method is mainly used to generate different concentrations of humidity and volatile compounds (ethanol, methanol, acetone, toluene, cyclohexane, etc.). The resulting mixture is then injected into the test chamber with the tested sensor. The volume of the test chamber is 50 mL. A Keithley 2400 sourcemeter was used to measure the resistance. A relay multiplexer is used to switch individual sensors, which is controlled by a National Instruments USB-6351 DAQ (Data Acquisition) device (16 analog inputs, 24 digital inputs/outputs, 2 analog outputs, maximum sampling rate 125 MS/s). The resistance values of the sensors are measured every 250 ms. The connection of the sensors between the multiplexer and the sourcemeter is performed using a coaxial cable to reduce interference from external electromagnetic fields. The VICI EUTA-4VL4MWE2 four-port two-way valve is used to switch the flow of gasses (test gas–purge gas) to ensure a rapid increase to the target concentration of the test gas, and to avoid the influence of overflows during switching. The apparatus and measurement process are controlled by the LabView application.

The change in sensor resistance (∆R/R_0_) as a function of exposure time was investigated. The response of the sensor is given by the change in relative resistance,

∆R/R_0_ = (R_g_ − R_0_)/R_0_, (1)

where R_g_ represents the resistances upon exposure to a specific gas and R_0_ is the reference resistances in synthetic gas. The fabricated sensors were used for the detection of ammonia (NH_3_), carbon dioxide (CO_2_), nitrogen dioxide (NO_2_), acetone (CH_3_COCH_3_), toluene (C_6_H_5_CH_3_), and humid air (RH) under various concentrations at room temperature.

## 3. Results and Discussion

### 3.1. Scanning Electron Microscopy (SEM) and Raman Spectroscopy

**Figure 6:**
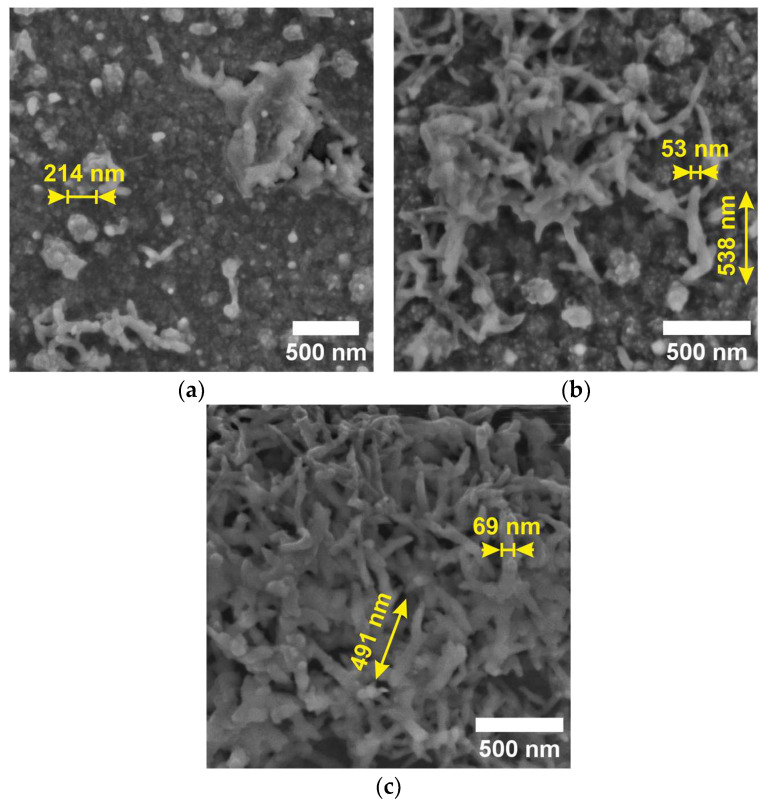
SEM micrographs of the deposited active layers surface morphology polymerized in: (**a**) aqueous solution, (**b**) sulfuric acid, and (**c**) acetic acid.

Scanning electron microscopy (SEM, TESCAN FERA3 GM) was used to analyze the surface morphology of the deposited active layers (Figure 6a–c). All active PANI layers have nanostructure morphologies, especially when polymerized in sulfuric acid solution and acetic acid solution. Polyaniline synthesized in an aqueous solution forms a granular form, while that synthesized in an acidic environment forms nanotubes. Polyaniline prepared in acetic acid exhibits the highest proportion of nanotubes.

**Figure 7:**
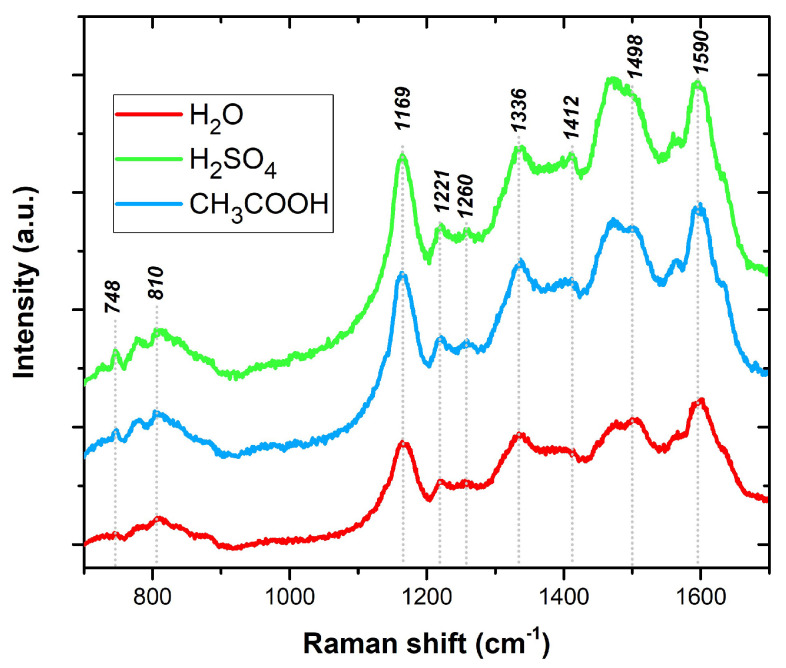
The Raman spectra of the PANI layers.

The PANI layers were also examined by Raman spectroscopy to confirm the deposited sensitive layers and their purity. Raman spectroscopy was performed at room temperature using a Renishaw inVia Qontor Raman spectrometer at a wavelength of 633 nm. The obtained spectra are shown in Figure 7. The spectrum of pristine PANI with main bands is described in Table 1 [23,24].

### 3.2. Temperature Analysis and Current-Voltage Characteristics of Polyaniline Layers

**Figure 8:**
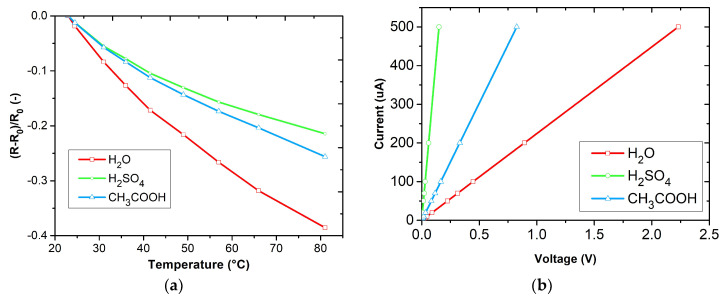
Temperature dependencies of sensitive layers (**a**) and current-voltage characteristics of sensitive layers (**b**).

The temperature dependencies of the prepared layers are shown in Figure 8a. The average values of the temperature coefficients of resistance (TCR) can be determined according to:

∆R/R_0_ = α·ΔT, (2)

where α is the temperature coefficient, ΔR is the difference in electrical resistance over a given temperature range, R_0_ is the initial temperature, and ΔT is the temperature range. Table 2 shows the average values of the temperature coefficients of the different sensitive PANI layers in a temperature range from 23 °C to 80 °C.

The temperature dependencies of the polyaniline layers exhibit a negative temperature coefficient. The polyanilines formed in acidic environments have a lower temperature dependence than the PANI formed in aqueous environments. The temperature dependencies show an exponential behavior, which is consistent with the temperature dependence of polyaniline described by the equation [25].

σ = σ_0_·exp(−(T_0_/T)^(1/d+1)^), (3)

where σ is the specific conductance, d is the dimension of the sample, σ_0_ and T_0_ are the parameters. If the sample is three-dimensional, we obtain the so-called Mott relation of temperature dependence, where the exponent is equal to:

1/d + 1 = 4(4)

The current-voltage characteristics shown in Figure 8b exhibit linear dependencies. The electrical resistance of the polyaniline layers at 25 °C and 50% relative humidity are shown in Table 3. It indicates that polyaniline formed in acidic media has lower electrical resistance than polyaniline formed in an aqueous solution.

### 3.3. Gas Sensing Analysis

**Figure 9:**
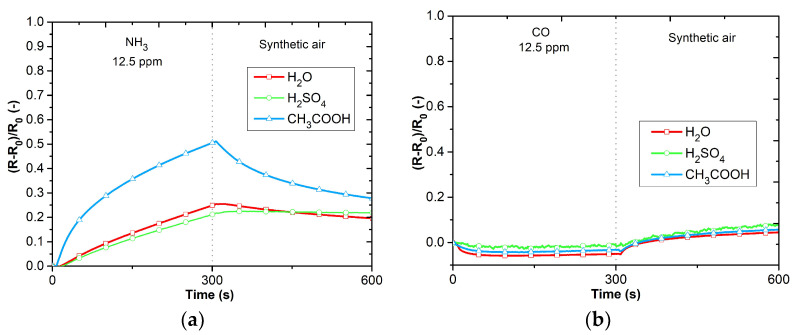

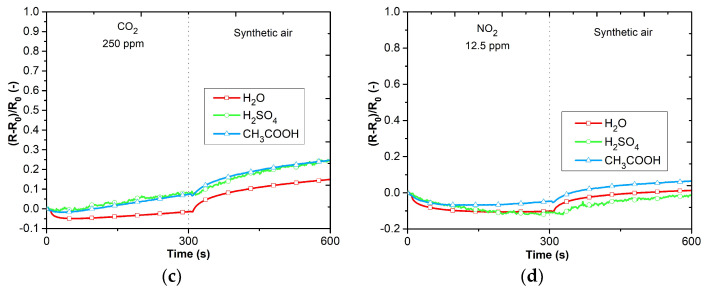
Responses of active layers: (**a**) reaction to NH_3_, (**b**) reaction to CO, (**c**) reaction to CO_2_, (**d**) reaction to NO_2_.

**Figure 10:**
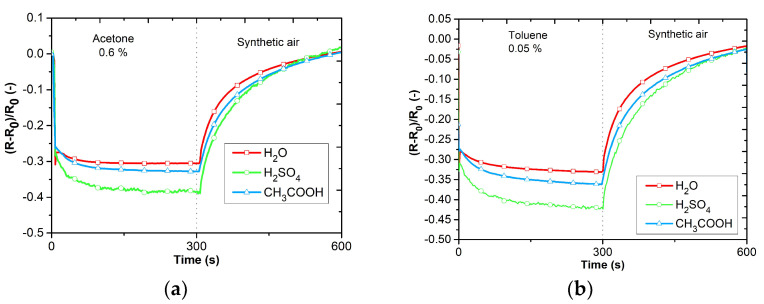
Responses of active layers toward VOCs: (**a**) reaction to acetone, (**b**) reaction to toluene.

**Figure 11:**
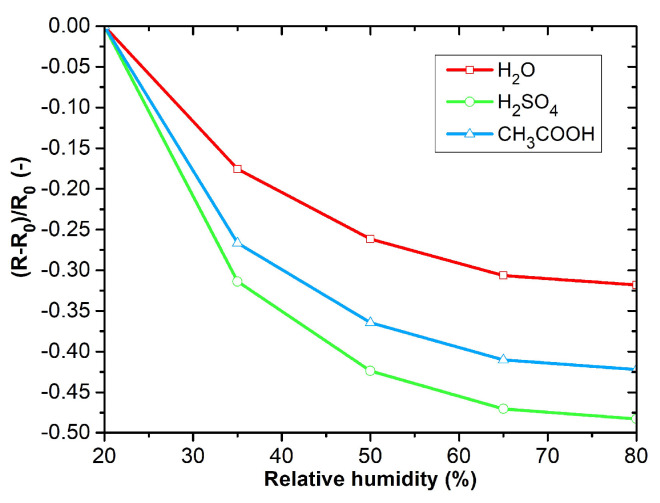
RH dependences of active layers.

The DC analysis was performed due to the expected use of the sensors in applications working with a DC power supply and the possibility of simple evaluation. The electrical resistance was measured as a function of the time changes in the gas concentrations. Figure 9a–d shows the responses of PANI layers toward ammonia (12.5 ppm NH_3_), carbon monoxide (12.5 ppm CO), carbon dioxide (250 ppm CO_2_), and nitrogen dioxide (12.5 ppm NO_2_). The sensitive layers were also tested with volatile organic compounds (VOCs), 0.6% of acetone, 0.05% of toluene (Figure 10a,b), and toward humidity (Figure 11). Three cycles with the specified concentrations (5 min of exposure to the test gas, 5 min of synthetic air (SA) purging, flow rate 100 mL∙s^−1^) were performed during the testing. The measuring current was set to 10 μA to reduce the possibility of heating the layers.

The active layers exhibit the highest sensitivity to ammonia (Figure 9a). Polyaniline polymerized in acetic acid exhibits the greatest sensitivity. The reactions to carbon monoxide, carbon dioxide, and nitrogen dioxide show low sensitivity (Figure 9b–d). Significant sensitivity can be observed to acetone, toluene, and humidity (Figure 10a,b and Figure 11). PANI layers exhibit a relatively fast response time to VOCs. Electrical resistance decreases with increasing relative humidity.

**Figure 12:**
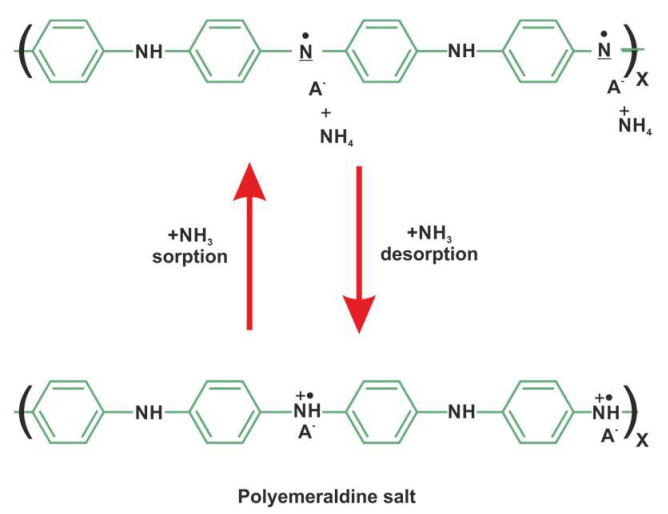
The reaction of PANI endowed with HA mineral acids (A=Cl, HSO_4_, ClO_4_, etc.) to ammonia [26].

Ammonia is a gas generally detected by PANI layers because the nitrogen atoms of both compounds play a similar role in forming coordination bonds with protons. The deprotonation/protonation mechanism is used to explain the sensitivity and reversibility of the mineral acid-doped PANI layer to ammonia (Figure 12). The free nitrogen doublet of the ammonia molecule can form a coordination bond with the free atomic orbital of the donating proton. This reaction leads to the deprotonation of the polyaniline nitrogen atoms, involving the removal of charge carriers (polarons) and an increase in electrical resistance. Moreover, it may participate in the gas swelling reaction of the polymer [26].

**Figure 13:**
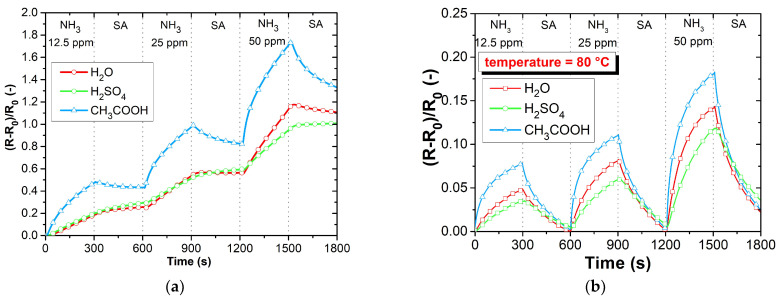
Gas characterization of active layers toward ammonia: (**a**) various concentrations at room temperature, (**b**) various concentrations at 80 °C.

**Figure 14:**
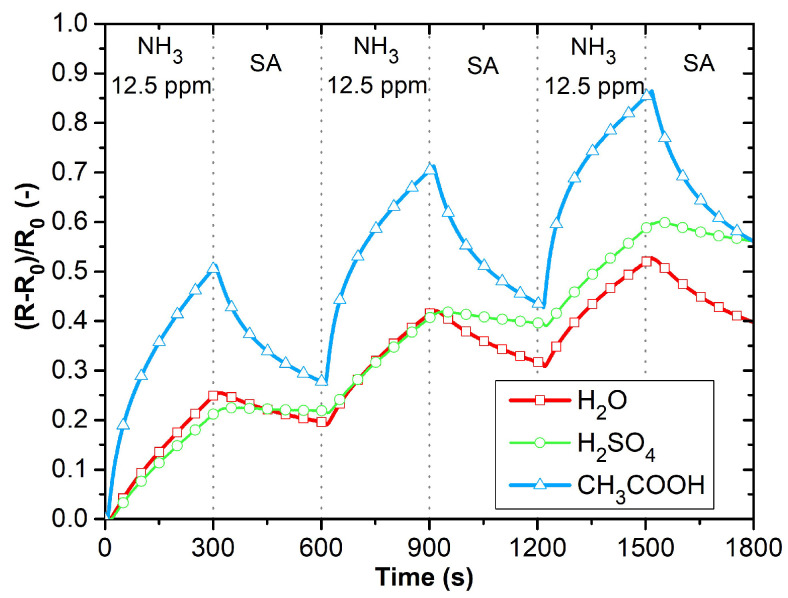
Gas characterization of active layers toward 12.5 ppm of ammonia.

Due to high sensitivity to ammonia, the sensing layers were further tested at both room temperature (Figure 13a) and at a higher temperature of 80 °C (Figure 13b) for various concentrations. When the layers are exposed to elevated temperatures up to 80 °C during testing with ammonia, a relatively good recovery of the sensor resistance to the initial value is observed. This can be explained by the better desorption of gas from the sensitive layer by supplying thermal energy. Figure 14 shows the response of the sensor layers to repeating the same ammonia concentration (12.5 ppm). The repeatability of the sensitive layer responses is observed from these waveforms.

**Figure 15:**
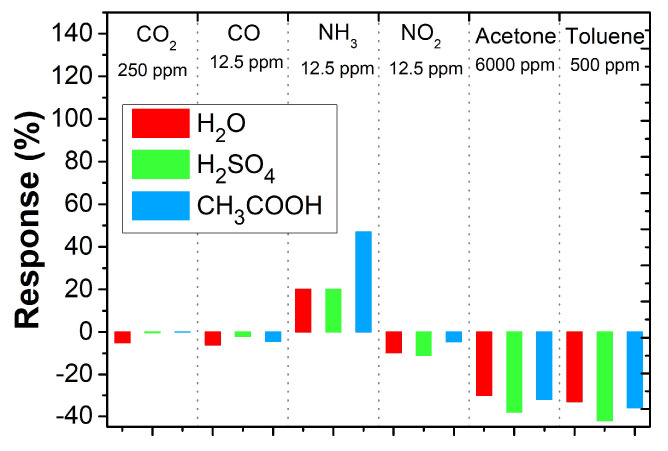
Overview of PANI layer response toward tested gasses and vapors.

Figure 15 summarizes the responses of the PANI layers to the tested gasses. High sensitivity is evident for ammonia, especially for PANI polymerized in acetic acid. Furthermore, it can be observed that ammonia is a reducing gas, while the other tested gasses have an oxidizing character. Similar reduction behavior was observed for chloroform [27] and hydrogen sulfide [28]. Increased sensitivity to higher concentrations of acetone and toluene can also be observed.

Our results were compared with similar works, as shown in Table 4. In [29], PANI doped with acrylic acid was synthesized and its production was described. The layer was exposed to various concentrations of ammonia from 1 ppm to 600 ppm. The sensor response was ΔR/R_0_ = 0.99 for a concentration of 58 ppm. Measurements were performed at room temperature. In [30], a sensor on a flexible substrate with a PANI sensitive layer was prepared. The authors used inkjet technology (modified Epson C46/C48 printer) for the deposition of PANI on the substrate. A response to an ammonia concentration of 50 ppm at 70 °C ΔR/R_0_ = 0.99 was achieved. Responses ΔR/R_0_ = 0.3 [31] and ΔR/R0 = 0.8 [32] were achieved using PANI doped with dodecylbenzenesulfonic acid. In [33], nanofibers doped with hydrochloric acid were created and the sensor response achieved ΔR/R_0_ = 2.9 at 50 °C.

## 4. Conclusions

In this paper, we demonstrated the fabrication and characterization of gas sensors with polyaniline active layers. These layers were polymerized in different polymerization solutions: aqueous, sulfuric acid, and acetic acid. Commercial Tesla Blatná platforms were used for sensors with polyaniline layers that were inserted into the polymerization solution during polymerization. These were coated with sensitive PANI layers. The sensor could be prepared with such sensitive layers in a single step, which is a significant benefit.

The surface morphology of these layers was examined using a scanning electron microscope. Polyaniline synthesized in an aqueous solution formed a granular form and in an acidic environment formed nanotubes. PANI layers polymerized in an acidic solution exhibited lower temperature sensitivity.

The polyaniline sensor was tested for the detection of CO, CO_2_, NH_3_, NO_2_, acetone, and toluene. The highest response was obtained for polyaniline polymerized in acetic acid toward ammonia (ΔR/R_0_ = 0.95 for 50 ppm of NH_3_). Better reversibility could be obtained at higher operating temperatures.

## Figures and Tables

**Table 1:** Values of the average temperature coefficients of the sensitive polyaniline layers in a temperature range from 23 °C to 80 °C.

Raman Shift (cm^−1^)	Description of Band
748	Q ring bending, C–C ring deformation
810	out-of-plane C–H vibrations in the aromatic rings
1169	C–H bending of the quinoid rings
1221	C–N in benzene diamine units
1260	C–N in benzene diamine units
1336	C–N^+^, a characteristic band of the polaron radical cation
1412	phenazine structures
1498	C=N of the quinoid nonprotonated diimine units
1590	C=C stretching vibration of the quinonoid ring

**Table 2:** Values of the average temperature coefficients of the sensitive polyaniline layers in a temperature range from 23 °C to 80 °C.

Sensitive Layer	α (K^−1^)
PANI polymerized in H_2_O	−0.0066
PANI polymerized in H_2_SO_4_	−0.0038
PANI polymerized in CH_3_COOH	−0.0044

**Table 3:** Electrical resistance values of sensitive polyaniline layers at 25 °C and 50 % relative humidity.

Sensitive Layer	R (Ω)
PANI polymerized in H_2_O	4470
PANI polymerized in H_2_SO_4_	306
PANI polymerized in CH_3_COOH	1660

**Table 4:** Comparison of implemented sensors with other works.

PANI Preparation Technology	Deposition Method of PANI	Substrate	Electrode Material	NH_3_ Concentration (ppm)	Operating Temperature (°C)	Relative Humidity (%)	Response ΔR/R_0_ (-)	Time Responses/Recovery (s)	Ref.
PANI acrylic -acid-doped	-	-	-	58	25	-	0.99	60/240	[29]
Ink-jet printing	Ink-jet	PET	Ag	50	70	-	0.15	100/200	[30]
PANI doped with dodecylbenzenesulfonic acid	Spin coating	Polyester (Tartan 950)	MWCNT ink	20	28	45	0.15	300/900	[31]
PANI doped with dodecylbenzenesulfonic acid	data	Al_2_O_3_	Au	50	25	Dry air	0.8	90/180	[32]
PANI nanofibers doped with HCl	Spin coating	Al_2_O_3_	Au	200	50	Dry N_2_	2.9	600/300	[33]
PANI prepared by polymerization in solution on a substrate	Coating during polymerization	Al_2_O_3_	Pt	50	25	Dry synthetic air	0.85	300/-	Our work

## Data Availability

Not applicable.

## Abbreviations

The following abbreviations are used in this manuscript:

DAQ	Data Acquisition
DC	Direct Current
ID	Interdigital
PANI	Polyaniline
PPy	Polypyrrole
PTh	Polythiophene
SA	Synthetic air
SEM	Scanning electron microscopy
VOC	Volatile Organic Compounds
